# Regulation of Three Nitrogenase Gene Clusters in the Cyanobacterium *Anabaena variabilis* ATCC 29413

**DOI:** 10.3390/life4040944

**Published:** 2014-12-11

**Authors:** Teresa Thiel, Brenda S. Pratte

**Affiliations:** Department of Biology, University of Missouri–St. Louis, St. Louis, MO 63121, USA; E-Mail: pratteb@umsl.edu

**Keywords:** mRNA processing, *Anabaena*, cyanobacteria, nitrogenase, V-nitrogenase, regulation

## Abstract

The filamentous cyanobacterium *Anabaena variabilis* ATCC 29413 fixes nitrogen under aerobic conditions in specialized cells called heterocysts that form in response to an environmental deficiency in combined nitrogen. Nitrogen fixation is mediated by the enzyme nitrogenase, which is very sensitive to oxygen. Heterocysts are microxic cells that allow nitrogenase to function in a filament comprised primarily of vegetative cells that produce oxygen by photosynthesis. *A. variabilis* is unique among well-characterized cyanobacteria in that it has three nitrogenase gene clusters that encode different nitrogenases, which function under different environmental conditions. The *nif1* genes encode a Mo-nitrogenase that functions only in heterocysts, even in filaments grown anaerobically. The *nif2* genes encode a different Mo-nitrogenase that functions in vegetative cells, but only in filaments grown under anoxic conditions. An alternative V-nitrogenase is encoded by *vnf* genes that are expressed only in heterocysts in an environment that is deficient in Mo. Thus, these three nitrogenases are expressed differentially in response to environmental conditions. The entire *nif1* gene cluster, comprising at least 15 genes, is primarily under the control of the promoter for the first gene, *nifB1*. Transcriptional control of many of the downstream *nif1* genes occurs by a combination of weak promoters within the coding regions of some downstream genes and by RNA processing, which is associated with increased transcript stability. The *vnf* genes show a similar pattern of transcriptional and post-transcriptional control of expression suggesting that the complex pattern of regulation of the *nif1* cluster is conserved in other cyanobacterial nitrogenase gene clusters.

## 1. Introduction 

Filamentous heterocyst-forming cyanobacteria fix atmospheric nitrogen to ammonium under oxic growth conditions. Nitrogen fixation occurs in specialized cells called heterocysts that differentiate at regular intervals in a filament in response to an environment that is deficient in fixed nitrogen [1,2,3,4]. Heterocysts, which comprise 5%–10% of the cells in a filament, have a glycolipid layer that may restrict oxygen diffusion into the cell, lack oxygen-evolving photosystem II activity, and have increased respiration, all of which serve to protect nitrogenase from oxygen [5,6,7,8,9]. *Anabaena variabilis* is unusual among the heterocyst-forming cyanobacteria in that it has three nitrogenases, which are expressed in cultures grown in different environmental conditions (reviewed in [10]). No other well-characterized cyanobacterial strain has three nitrogenases; in fact, no other characterized strain has even two. The primary nitrogenase that is expressed in cultures growing in an oxic environment that is deficient in fixed nitrogen but has adequate molybdate is the heterocyst-specific Mo-nitrogenase encoded by the *nif1* genes [11,12]. In an oxic environment that is low in fixed nitrogen and molybdate, but with vanadate, *A. variabilis* synthesizes an alternative, heterocyst-specific V-nitrogenase, encoded by the *vnf* genes [11,13]. The third nitrogenase, a Mo-nitrogenase, encoded by the *nif2* genes is made in vegetative cells only under anoxic growth conditions in an environment that is low in fixed nitrogen with molybdate [14,15,16]. Synthesis of all three nitrogenases is repressed in cells grown with a source of fixed nitrogen.

Nitrogenase activity, which requires the expression of at least a dozen genes, is found late in the differentiation process, after the heterocyst becomes microoxic [10]. The assembly of nitrogenase is a complex process requiring highly conserved proteins that are found in large *nif* clusters in all nitrogen-fixing bacteria. NifD (α-subunit) and NifK (β-subunit) are the two subunits of dinitrogenase, forming a heterotetrameric enzyme with two FeMo-cofactors [7Fe-9S-Mo-C-homocitrate] [17,18,19,20,21]. NifH, with a [Fe_4_-S_4_] cofactor, transfers electrons to the dinitrogenase [22]. NifS transfers sulfur from cysteine to NifU [23], which acts as a scaffolding protein for [Fe-S] cluster assembly [19,24]. The [Fe-S] clusters are transferred to NifB to make NifB-co, a [Fe_6_-S_9_] cluster that serves as the precursor to FeMo-cofactor [25,26]. NifE and NifN, a heterotetrameric complex with some similarity to NifD and NifK, respectively, function as a scaffold for FeMo-cofactor assembly, prior to its transfer to apo-nitrogenase [19,27]). NifW is thought to bind MoFe protein and to help with homocitrate processing [28]. NifX serves as a transient reservoir of FeMo-cofactor [29]. NifZ aids in P-cluster assembly [30,31] while NifV makes homocitrate, a component of FeMo-cofactor [19]. Missing in cyanobacteria are the genes for NifQ, the Mo donor to FeMo-cofactor [32], NifM, which stabilizes NifH [33,34], and NafY, which stabilizes the open conformation of apo-MoFe protein prior to the insertion of FeMo-cofactor [35,36]. NifP is a serine acetyltransferase that is thought to aid in expression of nitrogenase activity [37]. NifT/FixU is a very small, conserved protein that is found in *nif* clusters; however, its function is unknown [38,39]. In *Anabaena* sp. PCC 7120, NifJ, pyruvate-flavodoxin dehydrogenase is required for nitrogen fixation under iron-limiting conditions [40].

The alternative V-nitrogenase comprises two VnfD (α-subunit), *two* VnfK (β-subunit) and four δ-subunits, VnfG, forming a heterooctomeric enzyme with two FeV-cofactors [41,42,43]. Like NifH, VnfH, with a [Fe_4_-S_4_] cofactor transfers electrons to dinitrogenase. The V-nitrogenase shows different efficiency in substrate interactions than the Mo-nitrogenase; it is relatively inefficient in reducing dinitrogen and thus produces more hydrogen than the Mo-nitrogenase and, unlike the Mo-nitrogenase, it can reduce ethylene to ethane [44]. Because it is an inefficient nitrogenase and produces hydrogen, the V-nitrogenase of *A.* *variabilis* has been used to produce hydrogen in an outdoor bioreactor [45].

In the Proteobacteria, the *nif* genes are structured into multiple operons, including *nifHDK*, encoding the structural proteins of nitrogenase, *nifBQ*, producing the proteins required for FeMo-cofactor assembly, *nifUVSM*, whose products are needed for Fe-S cluster formation, and *nifENX*, encoding scaffolding proteins for the assembly of the nitrogenase complex [46,47]. In the Proteobacteria, *nif* genes are under the control of the NtrBC nitrogen regulatory system, which controls synthesis of the regulatory proteins, NifA and NifL [48]. Activation of *nif* genes in the absence of oxygen and fixed nitrogen requires NifA, as well as the alternative σ^54^ RNA polymerase [48]. Similarly, the *vnf* genes of *Azotobacter* *vinelandii* are controlled by the activator VnfA [49,50].

In nitrogen-fixing cyanobacteria there are no homologues of NtrBC, NifA or NifL and there is no homologue of VnfA in *A. variabilis*. The global nitrogen regulatory protein, NtcA, is required for nitrogen fixation in heterocyst-forming cyanobacteria; however, it is also required for heterocyst formation so its role in activation of nitrogen fixation genes is not yet known [4,51,52]. While no sigma factor specifically associated with nitrogen regulation, like the σ^54^ factor in Proteobacteria, has been identified in cyanobacteria, the sigma factor encoded by *sigE* is important, but not essential, for expression of the *nif* genes in *Anabaena* sp. PCC 7120 [53].

In *A. variabilis*, and in most nitrogen-fixing cyanobacteria whose genomes have been sequenced, the *nif* gene clusters comprise, in the same order, *nifB*, *fdxN*, *nifS*, *nifU*, *nifH*, *nifD*, *nifK*, *nifE*, *nifN*, *nifX*, and *nifW*. All *nif* clusters also have *hesAB* and *fdxH* as well as several conserved unidentified ORFs [54]. In *A. variabilis* and in *Anabaena* sp. PCC 7120, the *nifD* gene is interrupted by an 11-kb element that is removed from the chromosome of heterocysts by an excisase, XisA, late in heterocyst differentiation [55,56,57]. Transcription of the *nif* genes was first reported over 30 years ago [58,59]; however, little progress has been made in identifying key regulatory mechanisms. It has been assumed, based on Northern blot results and the assumed similarity to Proteobacteria, that the large *nif* cluster in cyanobacteria comprises several distinct operons: *nifB-fdxN-nifS-nifU* [58,60], *nifHDK* [59,61,62], as well as *hesAB* [63], *fdxH* [64] and, by default, *nifENXW*. In addition to the large conserved cluster, *nifP* is located just upstream of the *nifVZT* operon [39]. In *A.* *variabilis*, *nifP* is located about 11 kb downstream from the 3' end of the large *nif1* cluster. The best evidence for *nif* promoters in *Anabaena* are for those genes in which the apparent transcription start sites have been mapped. These include *nifB* [58,60,65,66,67], *nifH* [58,68], *hesA* [63,67], and *fdxH* [64]. Recent work from our lab that is described in more detail here has shown that there is a strong promoter driving *nifB1* and a separate promoter for *hesA1* in *A. variabilis*, but there is no promoter for *nifH1* or *fdxH1* [65,69]. While most of the expression of the large cluster of *nif1* genes in *A. variabilis* is driven by the *nifB1* promoter, there are additional weak promoters, including one in the *nifU1* gene and in the *nifE1* gene, that supplement transcription from *nifB1* [65,69]. While neither *nifH1* nor *fdxH1* has a promoter, the “transcription start sites” that were mapped upstream of these two genes are actually processed 5' transcript ends, not 5' primary transcription start sites [65,69].

## 2. Organization and Evolution of *nif*/*vnf* Gene Clusters in *A. variabilis*

**Figure 1:**
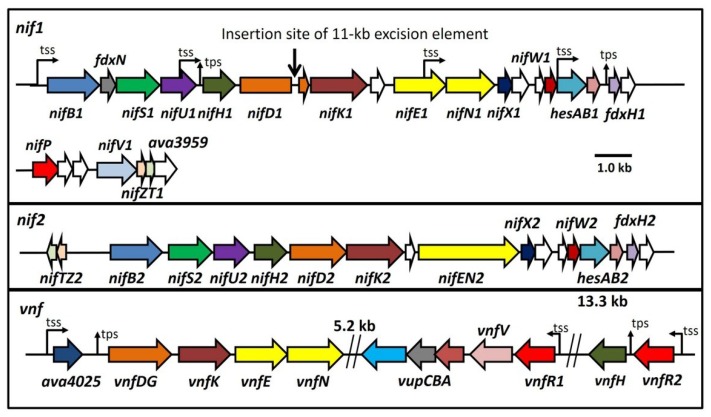
Maps of the three major nitrogenase gene clusters in *A. variabilis*. The 11-kb excision element in *nifD1* is not shown. tss, transcription start site; tps, transcriptional processing site. White ORFs indicate proteins of unknown function.

The organization of the three nitrogenase gene clusters of *A. variabilis* is shown in Figure 1 [11,16,70]. The nearest relative of the *nif1* cluster of *A. variabilis* is the sole *nif* cluster in *Anabaena* sp. PCC 7120; however, the *nif* cluster in *Anabaena* sp. strain PCC 7120 has a 55-kb excision element in *fdxN* that is not present in *A. variabilis* [71,72]. The *nif1* cluster is also very similar to the *nif* clusters in other heterocyst-forming cyanobacteria. In *Anabaena* spp., the nitrogenase encoded by these *nif1*-type genes is expressed only in heterocysts, even under anoxic growth conditions [16,73]. The similarly organized *nif2* cluster in *A. variabilis* is most similar in overall gene organization and gene similarity to the sole *nif* cluster of *Chroococcidiopsis thermalis* PCC 7203, a strain that belongs to a group of unicellular non-heterocystous cyanobacteria that grow in extreme environments and fix nitrogen only under anoxic conditions [74]. The similarity of the *nif2* gene cluster to the *nif* cluster in *Chroococcidiopsis thermalis* PCC 7203 is interesting, since the *Chroococcidiopsis* group is the closest relative of the heterocyst-forming cyanobacteria, based on 16S rRNA phylogeny [75]. In particular, the unusual fusion of the *nifE* and *nifN* genes into a single gene in the *nif2* cluster and in the *nif* cluster of *C.* *thermalis* suggests that these genes have a common ancestor. Another major difference between the *nif1* and *nif2* clusters in *A. variabilis* is the presence of an excision element only in the *nifD1* gene. That excision element is present in most, but not all, of the *nif* clusters in the genomes of sequenced heterocyst-forming cyanobacteria. Although the size of the element and the genes present in these excision elements varies among strains, all of them have a conserved excisase gene that removes the element during heterocyst differentiation, thereby restoring a complete *nifD* gene to produce the β-subunit of nitrogenase [55,72].

In contrast, the organization of the *vnf* genes that encode an alternative V-nitrogenase [11] is different from the two *nif* clusters, in part because synthesis and assembly of the V-nitrogenase depends on the products of some of the genes that make the Mo-nitrogenase, notably NifB, NifS, NifU and possibly several of the small proteins such as NifW, HesA, HesB and ferredoxins [76]. The *vnf* genes comprise *vnfDG*, a fusion of *vnfD* and *vnfG*, as well as genes *vnfK*, *vnfE* and *vnfN*. Unlike the *vnf* gene cluster in the Proteobacteria, there is no *vnfH* near the *vnfDGKEN* cluster, and in *A. variabilis*, *vnfH* is over 20 kb downstream from *vnfN*, with *vupABC* between *vnfH* and the other structural genes (Figure 1). Complete cyanobacterial genome sequences [77] have revealed strains that have genes very similar to the *vnf* genes of *A. variabilis* [70]. *Fremyella diplosiphon* UTEX 481 and *Fischerella muscicola* PCC 7414 have orthologs of *vnfDG*, *vnfK*, *vnfE* and *vnfN* as well as the vanadate transport genes, while *Fischerella* sp. PCC 9339 has orthologs of *vnfDG*, *vnfK*, *vnfE* and *vnfN* but is missing most of the vanadate transport genes. In contrast, *Chlorogleopsis* sp. PCC 7702 has orthologs for the vanadate transport genes, and has most of the structural genes for the V-nitrogenase; however, the fused *vnfDG* gene is missing the *vnfD* portion that encodes the α-subunit of the enzyme, which is essential for dinitrogenase activity. The presence of V-nitrogenase activity has not been confirmed in any of these strains.

## 3. Cell-Type Specific Expression of the Three Nitrogenases in *A. variabilis*

**Figure 2:**
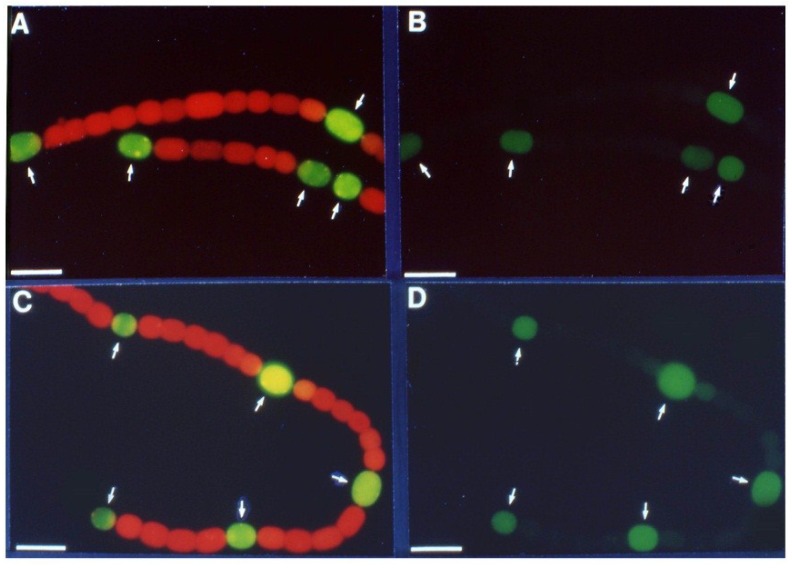
Oxic *vs.* anoxic expression of β-galactosidase in a *nifD1:lacZ* fusion strain. Cells grown in the absence of fixed N with oxygen; fluorescence from cleavage of fluorescein-β-d-galactopyranoside photographed without red cut-off filter (**A**); or with red cut-off filter (**B**); Cells grown in the absence of fixed N without oxygen; fluorescence from cleavage of fluorescein-β-d-galactopyranoside photographed without red cut-off filter (**C**); or with red cut-off filter (**D**). Arrows indicate heterocysts identified by bright field microscopy. Bar = 10 μM. Reproduced from [16] with permission.

In cyanobacteria, the best evidence for cell-type specific gene expression comes from imaging of cells expressing reporter genes such a *gfp*, *luxAB* or *lacZ* fused to cyanobacterial promoters. In our research, we have often used promoter:*lacZ* fusions because it is easy to assay β-galactosidase in the same cultures that are used for imaging and because there is no concern that the microoxic conditions in a mature heterocyst may affect the reporter protein, which might affect levels of GFP [78]. It was first shown in *Anabaena* sp. PCC 7120, using a Lux reporter, that the nitrogenase genes were expressed only in heterocysts even under anoxic conditions [73]. Similarly, in *A. variabilis* expression of *nifD1*:*lacZ* is confined to heterocysts, whether the cells are grown under oxic or anoxic conditions (Figure 2), indicating that some aspect of heterocyst development, possibly a heterocyst-specific activator, is required for *nif1* gene expression [16].

**Figure 3:**
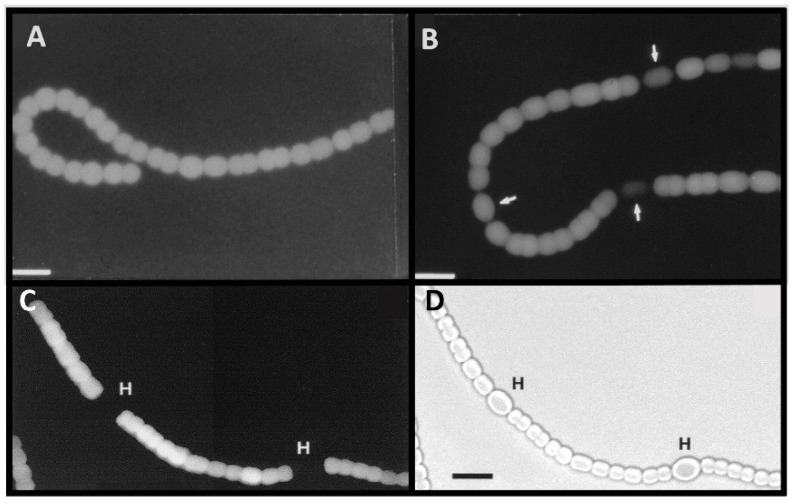
Anoxic β-galactosidase expression in a *nif2:lacZ* fusion strain. Panels (**A**) and (**B**): Cells with a *nifD2:lacZ* fusion were grown in the absence of fixed N, without oxygen; fluorescence from cleavage of fluorescein-β-d-galactopyranoside was photographed with red cut-off filter 6 h (A), or 24 h (B) after removal of fixed N. Arrows indicate heterocysts identified by bright field microscopy. Panels (**C**) and (**D**): Cells with a *nifD2:lacZ* fusion, grown for 48 h under oxic conditions without fixed N (to induce expression of the Nif1 nitrogenase) were then shifted to anoxic conditions for 4 h to induce expression of *nifD2*. Fluorescence from cleavage of fluorescein-β-d-galactopyranoside was photographed with a red cut-off filter (A). Light micrograph (D). Bar = 10 μM. Panels (A) and (B) are reproduced from [16] and panels (C) and (D) are reproduced from [15], with permissions.

In contrast, the *nif2* genes of *A. variabilis* are expressed only in cells grown under anoxic conditions and expression is evident within 4–6 h after nitrogen deprivation (Figure 3, panels A, C) [14,16]. The *nif2* genes are poorly expressed in the heterocysts that form under anoxic growth conditions, and the β-galactosidase activity seen in the heterocysts (Figure 3B) may reflect enzyme that was made in the vegetative cell prior to differentiation, rather than *de novo* synthesis in heterocysts. In support of this hypothesis, we observe that expression of the *nif2* genes is restricted to vegetative cells in filaments that are first grown under oxic conditions, to allow heterocysts to form, and then switched to anoxic conditions (Figure 3, panels C and D) [15].

**Figure 4:**
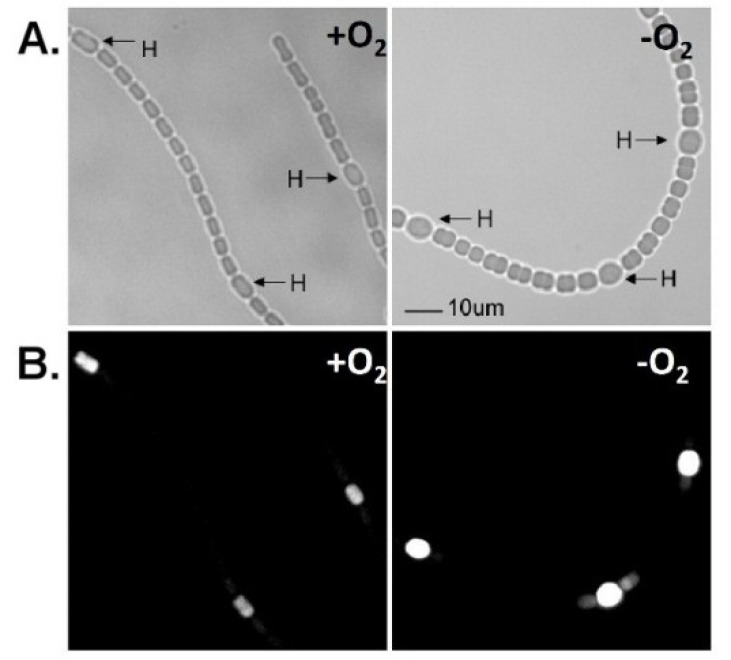
*In situ* localization of expression of *lacZ* under the control of the *vnfH* promoter. Strain BP272 (*vnfH:lacZ* fusion) was grown with fructose, in the absence of molybdate, with vanadate, under oxic (+O_2_) or anoxic (−O_2_) conditions. (**A**) Light micrographs; (**B**) Fluorescence from cleavage of fluorescein-β-d-galactopyranoside was photographed with a red cut-off filter. H = heterocysts. Bar = 10 µM. Reproduced from [15] with permission.

**Figure 5:**
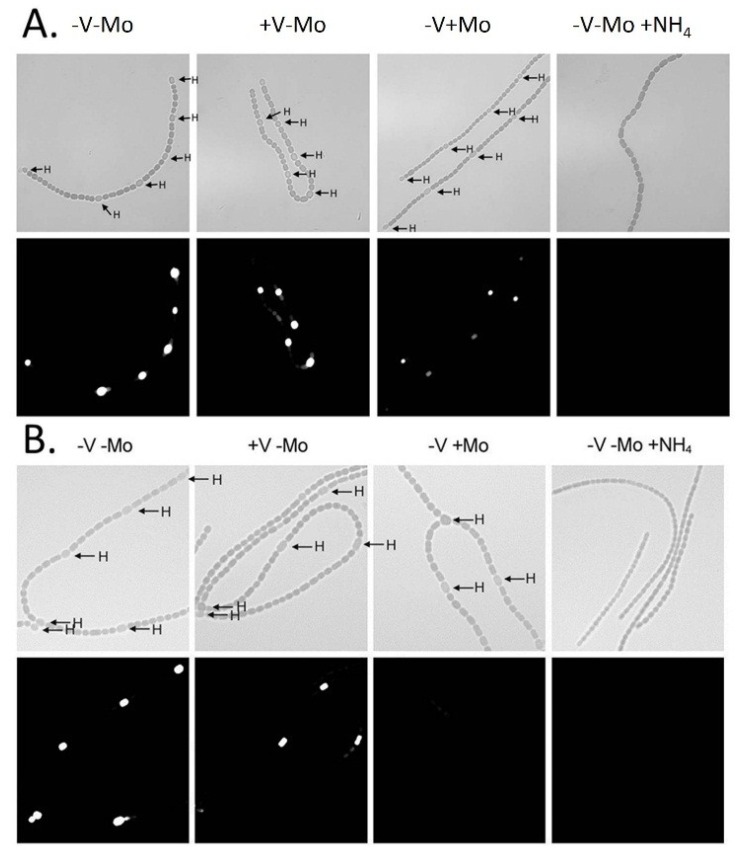
*In situ* localization of (**A**) *nifH1* expression and (**B**) *vnfDG* expression. Cells of strain BP221, with *lacZ* fused to the promoter region of *nifH1* (A), or BP193, with *lacZ* fused to the promoter region of *vnfDG*, were grown in AA/8 medium, with or without 1.0 μM molybdate or 1.0 μM vanadate or with 5.0 mM NH_4_Cl and 10 mM TES, pH 7.2. β-galactosidase activity was visualized using fluorescein-β-d-galactopyranoside. Top panels are bright field images showing heterocysts (H). Bottom panels show fluorescein fluorescence. Reproduced from [80] with permission.

**Figure 6:**
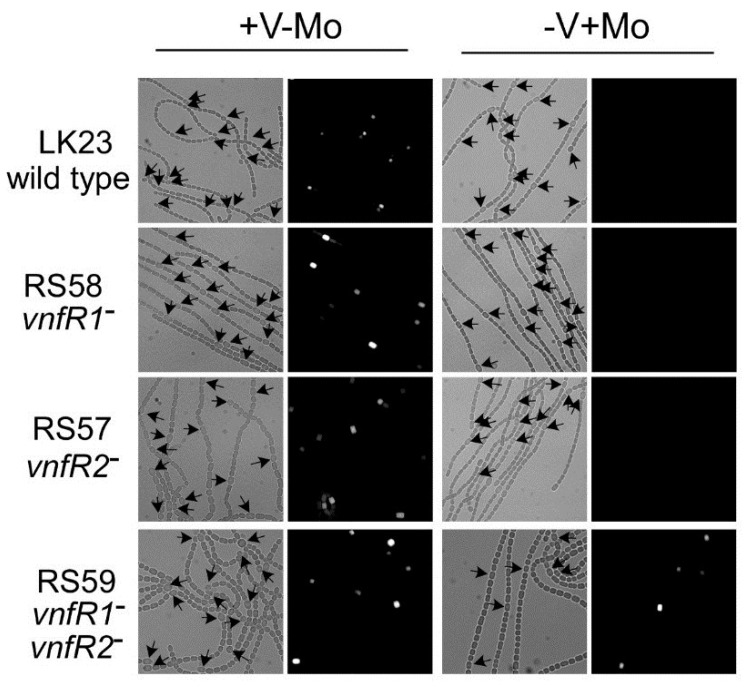
*In situ* localization of expression of *vnfDG*. The *vnfDG* gene was replaced in the chromosome by *lacZ*, in a *vnfR1* mutant (RS58), in a *vnfR2* mutant (RS57), in a *vnfR1* *vnfR2* double mutant (RS59) or in a wild-type background (LK23). β-galactosidase was visualized with a fluorescent substrate in cells grown with V (no Mo) or with Mo. Left panels are bright field images while the right panels are fluorescence images of the same field. Arrows indicate heterocysts. Reproduced from [81], with permission.

Expression of the *vnf* genes, like the *nif1* genes, is restricted to heterocysts in cells grown under oxic or anoxic conditions (Figure 4) [13], suggesting that, like the *nif1* cluster, expression of the *vnf* genes depends on a signal that is induced during heterocyst development; however, the *vnf* genes are not expressed unless the cells are starved for molybdate (Figure 5 and Figure 6) [79]. The fact that the V-nitrogenase requires NifB1 and possibly other gene products in the *nif1* cluster [76] is also consistent with the heterocyst-specific expression of the *vnf* genes.

## 4. Metal Transport and Its Effect on Nitrogenase Gene Expression

In many bacteria, including *Escherichia coli*, *Rhodobacter* *capsulatus* and *Azotobacter* *vinelandii*, high-affinity molybdate transport is mediated by an ABC-type transport system encoded by *modABC* genes [82,83,84,85]. ModA is the periplasmic component that binds molybdate, ModB is the transmembrane component of the permease, while ModC provides the energy from the cytoplasmic side of the membrane. Transcription of the *modABC* operon of *E. coli* is negatively regulated by dimers of ModE that are bound with four molecules of molybdate [86]. A high-affinity molybdate transport system in *A. variabilis*, with a *K_m_* for transport of molybdate of about 0.3 nM, is encoded by genes *modA* and fused genes *modBC* [79,87] that are located 2.7 Mb apart in the 6.36 Mb chromosome [70]. Mutants in this transport system cannot fix nitrogen unless molybdate is supplied at high concentrations (about 1 mM) or unless vanadate is supplied to allow assembly of the V-nitrogenase [79]. Cells starved for molybdate and vanadate express the *nif1* and *vnf* nitrogenase genes in heterocysts (Figure 5); however, because they cannot make nitrogenase, these nitrogen-starved cells produce a very high frequency of heterocysts and overexpress the nitrogenase genes. The addition of vanadate to Mo-starved cells has no effect on the expression of the *nif1* genes, while the addition of molybdate, which allows the Mo-nitrogenase to function, turns off expression of the *vnf* genes, but also decreases *nif1* gene expression and reduces heterocyst frequency compared to the Mo-starved cells (Figure 5). In contrast, the *vnf* genes are expressed only in the absence of molybdate, with or without vanadate [13]. NifH1, which is made in cells starved for molybdate, can substitute for VnfH in a *vnfH* mutant strain [13]. Further, in a strain with a *vnfH* promoter mutation that allows *vnfH* to be expressed in cells grown with molybdate, VnfH can substitute for NifH1 when that strain has a *nifH1* mutation. Thus, the two dinitrogenase reductases for the Mo-nitrogenase and the V-nitrogenase in heterocysts are able to function in place of each other suggesting that they are not involved in determining the metal specificity of these two nitrogenases [13]. This has not been shown *in vivo* for any other organism; however, using the nitrogenase for *A. vinelandii*, it has been shown *in vitro* that VnfH can replace NifH for the synthesis of the FeMo-cofactor and for maturation of the Mo-nitrogenase [88].

Between the *vnfDGKEN* and *vnfH* genes in the *A. variabilis* genome are the *vupABC* genes encoding the vanadate transport system that supplies vanadate for the V-nitrogenase [89] (Figure 1). The high-affinity vanadate transport system, with a K_m_ of about 3 nM is, to date, the only vanadate transporter that has been characterized. The vanadate transport genes, like the V-nitrogenase genes, are repressed by molybdate [89]. These genes are most similar to the tungstate transport genes of *Eubacterium* *acidaminophilum*. Similar genes are not present in the complete genomes of other bacterial strains that are known to have a V-nitrogenase, including *A. vinelandii, Rhodopseudomonas palustris,* and *Methanosarcina barkeri*, although the complete genome sequences of the cyanobacteria *Fremyella* *diplosiphon* UTEX 481, *Chlorogloeopsis* sp. PCC 7702, and *Fischerella* *muscicola* PCC 7414 have orthologs of the vanadate transport genes.

**Figure 7:**
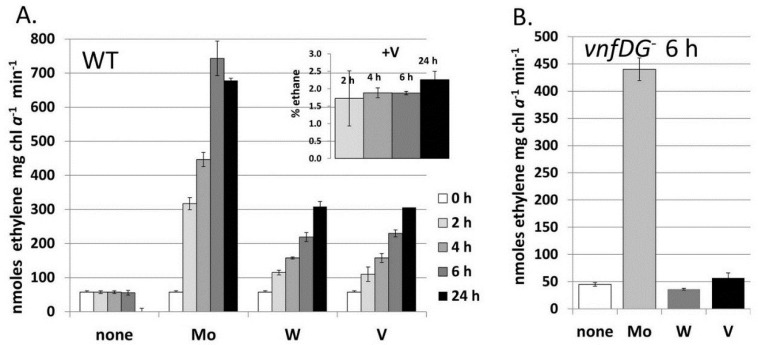
Metal-induced increase in nitrogenase activity in cells starved for molybdate and vanadate. *A. variabilis* strains FD (WT) and MB2 (*vnfDG* mutant) were grown in Mo- and V-free medium for at least 10 generation to deplete internal stores of these metals and then Na_2_MoO_4_, Na_3_VO_4_ or Na_2_WO_4_ (all at 100 nM) were added to these starved cells at 0 time. Acetylene reduction was measured for strain FD at 2 h, 4 h, 6 h and 24 h after metal addition (**A**) and for strain MB2 (*vnfDG* mutant) at 6 h after metal addition (**B**). The inset shows the ethane (% of ethylene) produced by strain FD with added Na_3_VO_4_, which were the only samples that produced any ethane. Reproduced from [80] with permission.

When *A. variabilis* is grown in a medium without fixed nitrogen and with less than 1.0 nM Mo and V, the cells become starved for both metals; however, slow growth continues, accompanied by low levels of nitrogen fixation [80]. This slow growth is abolished in a *nifDK1* mutant lacking the heterocyst-specific Mo-nitrogenase, but slow growth continues in a mutant lacking the V-nitrogenase, suggesting that only the Mo-nitrogenase is able to support slow growth in an environment with little molybdate or vanadate. Tungstate is transported by the molybdate transporter and could, theoretically, be incorporated into a nitrogenase [87]. The addition of tungstate, vanadate, or molybdate to cells starved for these metals resulted in an increase in nitrogenase activity, as measured by acetylene reduction, after two hours and this increase required new protein synthesis, suggesting that new nitrogenase was being synthesized with all these metals [80]. While tungstate functioned about as well as vanadate in supporting acetylene reduction, the cells to which tungstate was added did not grow any better with tungstate than with no added metal and did not produce ethane (Figure 7) [80]. A mutant lacking the V-nitrogenase showed no increase in nitrogenase activity upon addition of tungstate, suggesting that the V-nitrogenase, rather than the Mo-nitrogenase, was able to incorporate tungstate (Figure 7). Tungstate was able to substitute for molybdate in repressing transcription of a Mo-transport gene, but not the *vnfH* gene, which was, however, repressed by Mo [80]. This suggests that the Mo-dependent regulator of the molybdate transport system, probably the product of the *modE* homolog located just upstream of *modA* [79], interacts differently with molybdate/tungstate than the Mo-dependent regulators of the *vnf* genes, VnfR1 and VnfR2 [81] discussed in more detail below.

## 5. Transcription of Nitrogenase Genes

**Figure 8:**
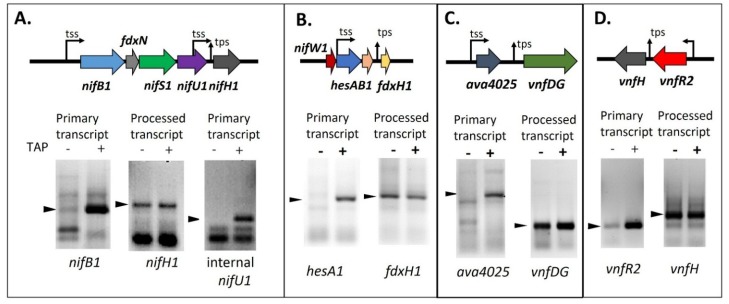
Determining the 5' ends of transcripts by 5' RACE with and without TAP treatment. (**A**) *nifB1* and *nifU1*, requiring TAP, are primary transcripts, but *nifH1* is a processed transcript; (**B**) *hesA1*, requiring TAP, is a primary transcript, but *fdxH1* is a processed transcript; (**C**) *ava4025*, requiring TAP, is a primary transcript, but *vnfDG* is a processed transcript; (**D**) *vnfR2*, requiring TAP, is a primary transcript, but *vnfH* is a processed transcript. tss, transcription start site; tps, transcriptional processing site. Arrows indicate the PCR products that were sequenced to determine the 5' transcript ends whose location is shown on the gene maps. Gel images in panels A and D are reproduced from [65], in panel B from [69], and in panel C from [81], with permissions.

By analogy with the *nif* operons of other nitrogen-fixing bacteria, including *Klebsiella* and *Azotobacter*, it has been thought that the large cluster comprising most of the *nif* genes in *Anabaena* could be divided into several discrete operons, including *nifBSU*, *nifHDK* and *nifENX*. Northern blots appeared to confirm this and, in fact, putative transcription start sites were mapped for the *nifB*, *nifH*, *hesA* and *fdxH* genes in the *nif* clusters of *Anabaena* spp. [58,60,63,64,65,66,67,68]. We mapped what appeared to be transcription start sites for *vnfDG* and *vnfH* (although they are actually processing sites) and confirmed that the apparent transcription start sites for *nifB1* and *nifH1* in *A.* *variabilis* were identical to those mapped in *Anabaena* sp. PCC 7120. We also identified additional weak promoters within the coding regions of *nifU1* and *nifE1* [65,69]. However, when we attempted to use the *nifH1* promoter to drive expression of *lacZ*, using a 300-bp promoter fragment that extended at least 150 bp upstream from the putative *nifH1* transcription start site, there was no reporter activity. A strain in which this same 300-bp fragment was used to drive expression of the *nifH1* gene failed to grow under nitrogen-fixing conditions and had no nitrogenase activity [90]. The same problem occurred when we attempted to drive transcription of *lacZ* with the putative *vnfDG* promoter [81] or with the *vnfH* promoter [65]; there was no expression using these promoter regions, although they extended well upstream from the putative transcription start sites. However, the promoters of other genes gave good activity, including *nifB1* [65], *ava4025*, the gene upstream of *vnfDG* [81], and *vnfR2*, the gene upstream of *vnfH* [65]. The answer to this puzzle came when the 5' ends of these transcripts were characterized and *nifH1*, *fdxH1*, *vnfH*, and *vnfDG* were found to have the 5' monophosphate end of a processed RNA, rather than the 5' triphosphate characteristic of a primary transcript (Figure 8) [65,69,81]. The difference in the 5' end structure of the mRNA can be determined by using a technique called RNA Ligase Mediated Rapid Amplification of cDNA Ends (5' RACE) with RNA that is treated, or not treated, with tobacco acid phosphatase (TAP), which converts a 5' triphosphate end to a 5' monophosphate end in preparation for ligation of the RNA adapter to the 5' end of the transcript. If the transcript is a primary transcript, the RNA adapter cannot ligate to the 5' triphosphate of the RNA unless it is treated with TAP; however, if the transcript is processed, it already has a 5' monophosphate and does not require TAP treatment. A 5' RACE product that is made equally well with RNA treated or not treated with TAP provides good evidence that the transcript is the result of processing *in vivo*. If very little 5' RACE product is made when the RNA is not treated with TAP, this indicates that the mRNA is a primary transcript. As shown in Figure 8, TAP treatment was not required to produce strong products by 5' RACE for *nifH1*, *fdxH1*, *vnfDG*, or *vnfH*, while TAP treatment was required for 5' RACE amplification of transcripts for *nifB1*, *nifU1*, *hesA1*, *ava4025* and *vnfR2* [65,69,81].

**Figure 9:**
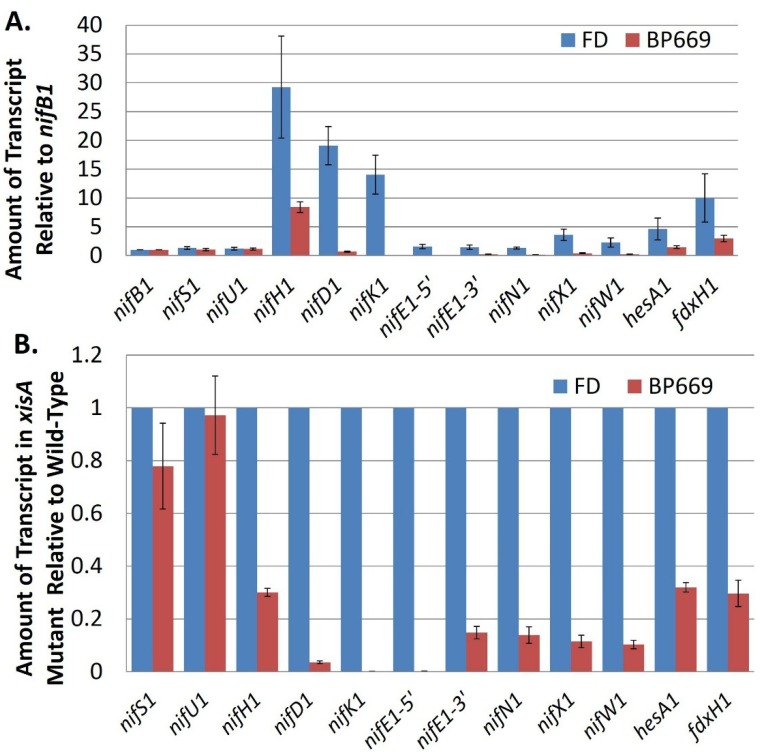
Transcript abundance of *nif* cluster genes in the wild-type strain compared to BP669, an *xisA* mutant. (**A**) The amount of transcript for genes in the *nif* cluster (relative to *nifB1*) was determined by RT-qPCR using RNA isolated from the wild-type strain, FD and from BP669, which cannot remove the 11-kb excision element in *nifD1*. Strains were grown with ammonium and then *nif* genes were induced by 24 h of starvation for fixed nitrogen; (**B**) Transcript levels in BP669 are shown relative to the wild-type strain, FD, in order to more clearly visualize the low levels of transcript for *nifK1*, *nifE1*, *nifN1*, *nifX1*, and *nifW1* in the mutant. Reproduced from [69], with permission.

**Figure 10:**
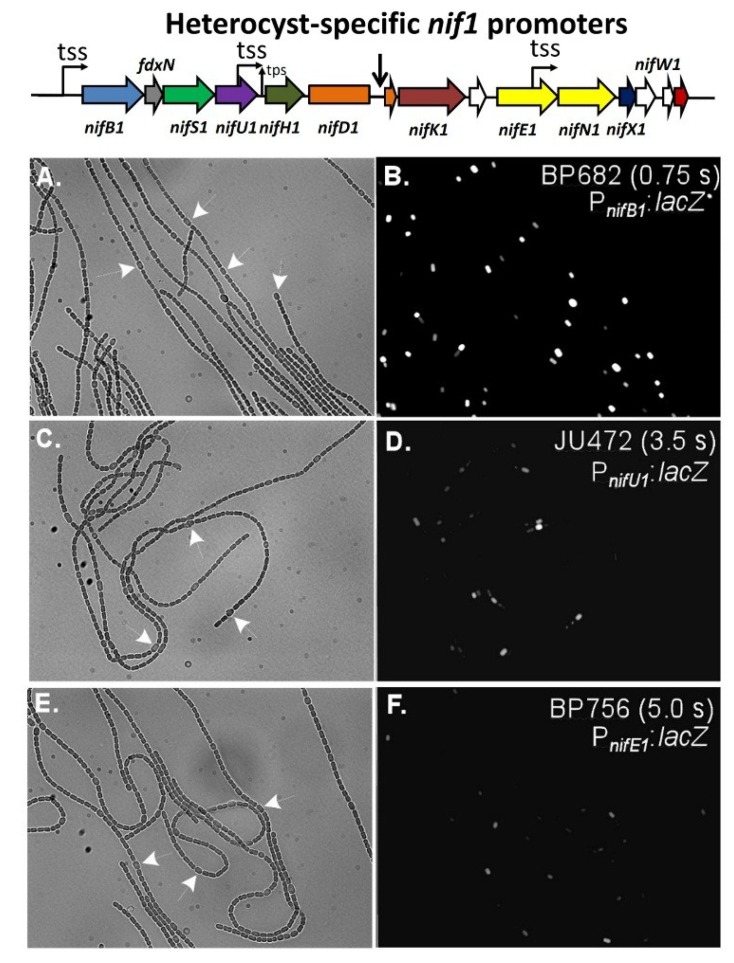
*In situ* localization of expression of β-galactosidase in strains with fusions of a promoterless *lacZ* to the *nifB1* promoter alone in strain BP682 (panels (**A**) and (**B**)), the *nifU1* promoter alone in strain JU472 (panels in (**C**) and (**D**)), or the *nifE1* promoter alone in strain BP756 (panels (**E**) & (**F**)). Panels (A), (C), and (E), are bright-field images of filaments with white arrows indicating a few representative heterocysts. Panels (B), (D) and (F) are fluorescence images showing the expression of β-galactosidase primarily in heterocysts. Exposure times in seconds for the fluorescence images are provided in panels (B), (D) and (F). Reproduced from [69], with permission.

Transcription of the *nif1* gene cluster of *A. variabilis* appears to depend primarily on the promoter for the first gene in the cluster, *nifB1*. While there is a promoter inside *nifU1*, it is very weak compared to the *nifB1* promoter and a strain in which *nifH1* is driven only by the *nifU1* promoter fixes nitrogen poorly compared to the wild-type strain [65]. There is no promoter upstream of *nifK1* or *nifE1*, so their transcription depends on the *nifB1* and *nifU1* promoters. Like the *nifU1* promoter, the promoter within *nifE1* is weak, suggesting that it serves an auxiliary rather than a primary function for gene expression [69]. Further support for the importance of the *nifB1* promoter in expression of the far downstream genes, including *nifKENXW1* is the near loss of these transcripts in a mutant strain that lacks *xisA*, the gene that makes the excisase that removes the 11-kb element from *nifD1* [69]. In this mutant, the *nifB1* and *nifU1* promoters cannot drive expression of the genes downstream from the 11-kb element, which is not excised, and these genes are poorly transcribed (Figure 9). Even *hesA1*, which has its own promoter, shows decreased expression in the *xisA* mutant, which suggests that the *nifB1* promoter is capable, at least partially, of driving transcription of a gene that is 14 kb away. Consistent with the fact that the *nif1* cluster encodes an enzyme that functions only in heterocysts, the *nifB1*, *nifU1* and *nifE1* promoters showed heterocyst-specific expression (Figure 10) [69].

The primary promoter for the structural genes for the V-nitrogenase, *vnfDGKEN*, is the Mo-repressible promoter for the gene upstream from this cluster, *ava4025*. The predicted product of this gene shows similarity to the periplasmic component of molybdate transporters, suggesting that it may have a role in sensing molybdate in the environment; however, a mutant in *ava4025* has no apparent phenotype and the gene is not required for Mo-repression of *vnfDGKEN* [81]. Although the *ava4025* promoter controls expression of *ava4025* and *vnfDG*, levels of *vnfDG* transcript are about 500-fold higher than *ava4025*, perhaps resulting from increased stability of the *vnfDG* transcript, which is processed at the site that was initially identified as the transcription start site [11,81]. Expression of *vnfDG*, under the control of the *ava4025* promoter is heterocyst specific (see Figure 5 and Figure 6) [81]. Like *vnfDGKEN*, *vnfH*, encoding the dinitrogenase reductase component of the V-nitrogenase, is the result of the processing of a transcript that is made from the promoter of the upstream gene, *vnfR1*. Although we initially reported, based on Northern blots, that vanadate transport genes, *vupABC*, form an operon [89], it now seems possible that the promoter for *vnfR1*, located upstream of the *vupABC* cluster may control these genes as well as the gene between *vnfR1* and *vupABC*, which may be *vnfV*, and that the *vupABC* transcripts may also result from RNA processing; however, this hypothesis awaits experimental support.

**Figure 11:**
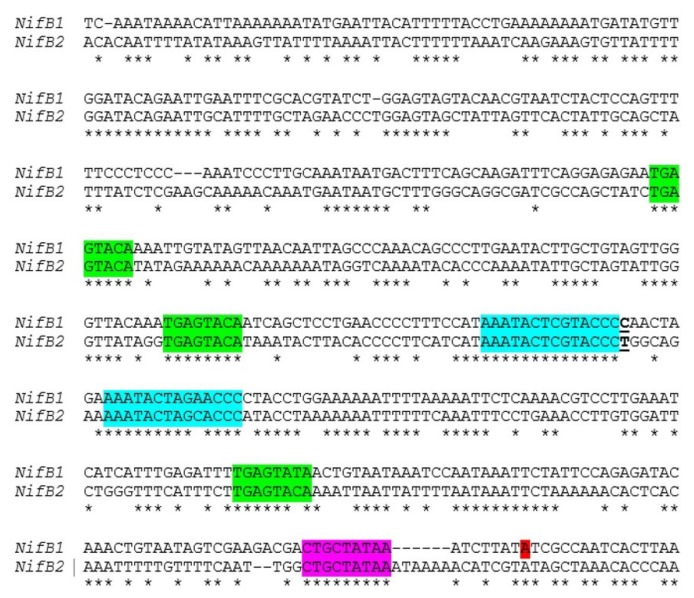
Similarity of the *nifB1* and *nifB2* regions upstream from the promoter. Alignment of the region upstream of the transcription start site of *nifB1*, shown in red, with a similar region upstream of *nifB2*. A putative extended −10 region is highlighted in magenta, a conserved TGAGTATA motif is highlighted in green, and another conserved motif is highlighted in turquoise. The T that is underlined at the end of the first motif in turquoise indicates the fusion site of a *nifB2*:*nifB1* hybrid promoter (see Figure 12).

**Figure 12:**
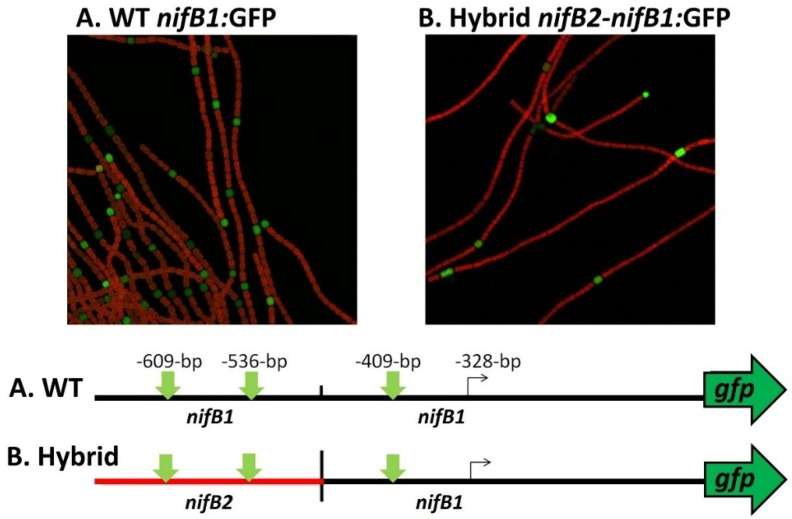
Heterocyst-specific expression of the *nifB1* promoter and a *nifB2*:*nifB1* hybrid promoter. The wild-type *nifB1* promoter was fused to the reporter gene, *gfp*, and a hybrid promoter of *nifB2*/*nifB1*, fused at the first nucleotide following the first conserved motif shown in turquoise in Figure 10, was also fused to *gfp* using fusion PCR, and the construct was integrated into the *A. variabilis* genome as described previously [65,81]. Strains were imaged by confocal microscopy 24 h after nitrogen step-down. GFP fluorescence was localized to heterocysts, which are green in the images. The map of the fusion shows the location of the tss in *nifB1* at −328 bp from the translation start site and the approximate location of the putative tss of *nifB2*. The vertical green arrows on the map show the locations of the three conserved motifs that are highlighted in green in Figure 11.

Little is known about the control of *nif* genes that function under anoxic conditions, including the *nif2* cluster in *A. variabilis*; however, the conservation of the organization of the entire cluster suggests that these genes may also be under the control of a single primary promoter. There are striking similarities in the sequences of *nifB1* and *nifB2* in the region upstream of the *nifB1* transcription start site, including conserved motifs, that suggest that the two *nifB* genes have some aspects of regulation in common (Figure 11). To test this hypothesis we created a fusion between the upstream region of *nifB2*, up to and including the first conserved motif shown in turquoise (Figure 11), to the downstream region of *nifB1* and then fused the hybrid promoter to GFP (strain JJ146). Expression of the *nifB2*:*nifB1* hybrid promoter fused to GFP was localized specifically to heterocysts as was GFP expressed from the control *nifB1* promoter (strain JJ72) (unpublished data and Figure 12). This heterocyst-specific expression of the hybrid promoter indicates that the conserved region upstream of the promoter of *nifB1* may serve in regulation that senses oxygen levels, since that signal is the primary one that induces expression of *nifB2*, and that heterocyst specificity may be conferred by sequences closer to the promoter that are not shared with the *nifB2* promoter and by heterocyst-specific protein(s) that may recognize these sequences. An understanding of the roles of the various elements in the promoter and upstream regions awaits a more detailed genetic analysis of both promoters and the identification of proteins that may control cell-specific transcription of the different nitrogenase genes.

The lack of discrete operons in the *nif1* clusters is inconsistent with the differences in transcript levels for different *nif* genes, especially for the highly abundant *nifH1*. The *nifH1* transcript is present in much greater quantity than *nifU1*, the gene directly upstream of *nifH1*, and the other structural genes for nitrogenase, *nifD1* and *nifK1*, also show high levels of transcription compared to *nifB1* (Figure 9A). If a strong promoter is not directly driving transcription of *nifHDK1*, then the higher levels of these transcripts may result from their stability. A striking feature of the region near the transcriptional processing sites of *nifH1*, *vnfH1*, *vnfDG*, and *fdxH1* is the presence of conserved stem-loop structures that may play a role in stabilizing the transcript [65,69]. By measuring RNA by RT-qPCR at various times after the addition of rifampin, which inhibits initiation of transcription by RNA polymerase, we determined the half-lives of most of the genes in the *nif1* gene cluster [69]. The half-life of *nifH1* is much longer than the genes upstream of the processing site and the half-lives of the transcripts downstream of *nifH1* decline with increasing distance from the processing site [69] (Table 1), suggesting that transcript stability plays a major role in controlling the relative amount of transcript. A mutant strain in which the stem-loop structure located at the processing site of *nifH1* is abolished shows a shorter half-life for the *nifH1* transcript than the wild-type strain and nitrogen fixation is strongly inhibited in this mutant (Thiel; unpublished data).

**Table 1:** Half-life of *nif1* transcripts ^1^.

Gene	Half-Life (min)
*nifB1*	12.3 ± 2.2
*nifS1*	14.7 ± 4.8
*nifU1*	8.6 ± 2.3
*nifH1*	33.8 ± 7.8
*nifD1*	22.2 ± 4.1
*nifK1*	16.7 ± 3.6
*nifE1*	7.4 ± 1.3
*nifN1*	9.1 ± 2.5
*nifX1*	12.3 ± 2.1
*hesA1*	20.9 ± 4.6
*fdxH1*	20.5 ± 4.0

Note: ^1^ Data are taken from [69], with permission.

## 6. Proteins Involved in Regulation of Nitrogenase Genes

The global regulator NtcA affects many genes that respond to nitrogen availability in the cell and its binding sites have been the subject of several studies [52,91,92]. Although NtcA is a regulator in all cyanobacteria, its significance in heterocyst-forming cyanobacteria is the key role it plays in sensing nitrogen starvation and initiating the complex process of heterocyst differentiation [3,4]. In addition to its role in activating genes that are required for the differentiation of heterocysts, NtcA activates expression of PipX, which is thought to work in concert with NtcA to allow full expression of late heterocyst-specific genes, including *nifH*, *coxB3* and *coxB2* (encoding heterocyst-specific cytochrome oxidases). A *pipX* mutant shows low levels of expression of these genes and is impaired in nitrogenase activity [93,94]. However, since the expression of the *nif* operon depends on low oxygen levels that result from high respiration that is mediated by the products of the *cox2* and *cox3* genes, the low levels of *nif* gene expression may be a secondary effect of relatively high oxygen levels in the heterocyst resulting from low levels of *cox* gene expression in the *pipX* mutant. In *Anabaena* sp. PCC 7120, *patB* was shown to be important for growth in the absence of fixed nitrogen [95] and we have found that expression of *nifB1* requires PatB1 and expression of *nifB2* requires PatB2 (Thiel; unpublished data). Similarly, in a non-heterocystous cyanobacterium a PatB homologue called CnfR has been identified as a key regulator of *nif* gene expression [96].

NtcA has been reported to bind weakly to a region upstream of *nifH* in *Anabaena* sp. PCC 7120 and a putative non-canonical NtcA binding site was identified [68,97]. However, recent ChIPSeq data for *Anabaena* sp. PCC 7120 showed that NtcA did not bind to any region upstream of *nifH*, but rather to a site within the coding region of *nifH* in *Anabaena* sp. PCC 7120 [52]. In this study NtcA was found to bind upstream of *nifB*, but the binding site was hundreds of nucleotides upstream of the transcription start site of *nifB* [52], suggesting that NtcA does not directly control expression from the *nifB* promoter. Using the *nifUH1* intergenic region of *A. variabilis* as the target, we were unable to detect binding of NtcA and mutations in the putative NtcA binding site in this region had no effect on expression of *nifH1* [65]; however, this is not surprising since we could find no evidence of a promoter in the *nifUH1* intergenic region (see Section 5 above). There may be differences in *nifHDK* regulation between *Anabaena* sp. PCC 7120 and *A. variabilis*, and the putative NtcA binding site upstream of *nifH* in *Anabaena* sp. PCC 7120 is not well conserved in *A. variabilis*. However, because of the high degree of overall sequence homology between the two *nif1* clusters in both strains, it seems unlikely that the same 5' *nifH* transcript end, found in both strains, results from fundamentally different processes. Further, a RNAseq mapping technique that identified transcription start sites (and excluded processed sites) for the genome of *Anabaena* sp. PCC 7120 found the anticipated *nifB* transcription start site at the published site, but failed to identify the putative *nifH* transcription start site, even though it is found at levels of at least 20-fold higher that *nifB* [67]. It is clear that NtcA is important for expression of the *nif* genes, but its effect is likely to be indirect, reflecting the fact that it may be essential for the expression of other genes whose products may act more directly to regulate expression of the *nif* genes.

The role of NtcA in expression of the *nif2* cluster is also not clear. An *ntcA* mutant of *A. variabilis* failed to fix nitrogen using the *nif2*-encoded Mo-nitrogenase, indicating that NtcA has an important role in expression of this enzyme; however, there is no canonical NtcA binding site, GTAN_8_TAC, anywhere in the region that is likely to have the promoter [15]. Because of the diversity of NtcA binding sites and of their locations relative to the start of genes [52] as well as the global effect of NtcA regulation it may be difficult to assign a specific role for NtcA in expression of these nitrogenase genes.

There are at least two proteins that are repressors of *vnfDG*, VnfR1 and VnfR2. VnfR1 is encoded by *ava4042*, located upstream of the vanadate transport genes, *vupABC* (Figure 1) [89]. VnfR2 is encoded by *ava4055*, upstream from *vnfH*, which encodes the dinitrogenase reductase for the V-nitrogenase. The promoter for *vnfR2* serves both *vnfR2* and *vnfH* (Figure 8) [65]. These proteins with a conserved *N*-terminal helix-turn-helix motif show 73% protein identity and act as Mo-dependent repressors that independently repress transcription of *ava4025*-*vnfDG* in cells grown with molybdate [81]. Although each protein can repress expression of *ava4025*-*vnfDG*, only VnfR1 binds specifically, *in vitro*, to a region upstream of the *ava4025* promoter. Cells lacking either *vnfR1* or *vnfR2* still show heterocyst-specific, Mo-repressed expression of *ava4025*-*vnfDG*. A mutant lacking both *vnfR1* and *vnfR2* expresses *ava4025*-*vnfDG* in the presence of Mo and expression is heterocyst specific (Figure 6), indicating that other factors activate expression of this promoter in heterocysts. 

## 7. Conclusions

Although there are three nitrogenases in *A.* *variabilis*, and two of them are Mo-nitrogenases, the tight control of expression of the *nif1*, *nif2* and *vnf* genes ensures that the cell makes the correct enzyme for the environment in which it is growing. This regulation includes differential expression of nitrogenases in response to cell differentiation, oxic *versus* anoxic growth conditions, and for environments with or without molybdate. The *nif2* cluster has not been found in any other well-characterized heterocyst-forming cyanobacterium and it shares an evolutionary origin with *nif* genes from non-heterocystous cyanobacteria especially with the primitive cyanobacterium *Chroococcidiopsis thermalis* [75,98], suggesting that an ancestor of this unicellular cyanobacterium may represent the evolutionary origin of the *nif2* genes. The *vnf* genes are not present in well-studied cyanobacteria; however, these genes have recently been found in cyanobacteria in a lichen symbiosis [99], suggesting that they may be represented in symbiotic interactions. The culturable cyanobionts from the water fern *Azolla filiculoides*, *Anabaena* spp. [58,100], are virtually indistinguishable morphologically and physiologically from *A. variabilis* and also have *vnf* genes [11] and *nif2* genes [16]; however, the non-culturable *Azolla* symbiont, *Nostoc* *azollae* 0708, shows a degraded genome incapable of supporting independent growth and this strain lacks both *vnf* and *nif2* genes [101].

While we understand at an environmental-response level and even at a whole-cell level how these three nitrogenases are regulated, information at the molecular level is still lacking. The fact that at least two of the three gene clusters employ RNA processing, and its associated transcript stability, as a regulatory mechanism suggests that this may be a more general mechanism of cyanobacterial gene regulation; however, that needs to be tested experimentally. While several promoters that show late heterocyst-specific gene expression have been identified, including a number that are described here, we still do not understand how those genes are activated late in heterocyst development. Proteins NtcA [4] and SigE [53] are important for expression of late heterocyst genes, but their specific function in controlling these genes is not known. Research is still needed to understand how the environmentally important process of nitrogen fixation and the synthesis of associated essential proteins, such as the uptake hydrogenase, ferredoxins, and cytochrome oxidases, are regulated.
